# In Vivo Study of *Moringa oleifera* Seed Extracts as Potential Sources of Neuroprotection against Rotenone-Induced Neurotoxicity

**DOI:** 10.3390/plants13111479

**Published:** 2024-05-27

**Authors:** Chand Raza, Sehrish Mohsin, Mehwish Faheem, Uzma Hanif, Hamad Z. Alkhathlan, Mohammed Rafi Shaik, Hasib Aamir Riaz, Rabia Anjum, Husna Jurrat, Merajuddin Khan

**Affiliations:** 1 Department of Zoology, Government College University, Lahore 54000, Pakistan; 2 Department of Botany, Government College University, Lahore 54000, Pakistan; 3 Department of Chemistry, College of Science, King Saud University, P.O. Box 2455, Riyadh 11451, Saudi Arabia; khathlan@ksu.edu.sa (H.Z.A.); mrshaik@ksu.edu.sa (M.R.S.); 4 Department of Molecular Biology, Cell Biology and Biochemistry, Brown University, Providence, RI 02912, USA

**Keywords:** Parkinson’s disease (PD), rotenone, *Moringa oleifera* seeds, motor deficits

## Abstract

Parkinson’s disease (PD) is a leading neurodegenerative disorder affecting 1–3 percent of the elderly population. Oxidative stress is the primary factor for the neurodegeneration of Substantia Nigra (SN). The current study aims to assess the seed extracts of *Moringa oleifera* (MO) on rotenone-mediated motor function impairments in a PD mouse model. For this purpose, two different seed extracts of MO were prepared, including aqueous MO (AqMO) and ethanolic MO (EthMO). Male Swiss albino mice were grouped into five groups. Mice received 2.5 mg/kg rotenone for 21 consecutive days, and control mice received the vehicle. Extract-treated mice received 200 mg/kg AqMO and EthMO separately, orally and daily for 28 days. Sinemet-treated mice received 20 mg/kg, oral dose, as a positive group. The motor function performance was evaluated using standard neurobehavioral tests. The antioxidant potentials of MO seed extracts were estimated by lipid peroxidation (LPO), reduced glutathione (GSH), glutathione-s-transferase (GST) and catalase (CAT) activities in mice brain homogenates. The PD mice brain SN sections were investigated for neurodegeneration. MO seed extract-treated mice showed a significant reduction in motor dysfunction compared to rotenone-treated mice as assessed through the open field, beam walk, pole climb-down, tail suspension, stride length and stepping tests. Increased antioxidant capacities of the PD mice brains of MO extract-administered groups were observed compared to the control. A histological study showed reduced signs of neurodegeneration, vacuolation around multipolar cells and cytoplasmic shrinkage in MO extract-treated mice SN brain sections. Collectively, MO seed extracts protected the animals from locomotor deficits induced by rotenone, possibly through antioxidant means, and seem to have potential applications in neurodegenerative diseases.

## 1. Introduction

Parkinson’s disease (PD) is a neurodegenerative disease characterized by the loss of dopaminergic neurons of Substantia Nigra (SN) and affects 1–3% of the population. It leads to progressive loss of motor functions and includes resting tremor, bradykinesia, postural instability and rigidity [1]. Oxidative stress is among the leading causes of PD features with dopaminergic (DA) neuron loss, neuroinflammation and dysfunctional mitochondria [2]. The natural production of reactive oxygen species (ROS) and clearance through enzyme-mediated antioxidant capacities operate in normal homeostasis. However, augmented ROS generation and perturbed antioxidant capacities have devastating effects on DA neurons. Oxidative stress-induced degeneration of DA neurons of SN results in the inadequate release of dopamine [3]. Dopamine is the principal neurotransmitter mediating motor activities from mid-brain basal ganglia, and a marked reduction in its release is observed in PD patients compared to age-matched healthy controls [4]. A leading cause of sporadic PD is mitochondrial dysfunction, particularly impairments in processes involved in energy production, such as the electron transport chain [5].

Although PD is not a fatal disease, it greatly affects the quality of life of affected persons and leads to huge socio-economic burdens [6]. Current treatment and management strategies for this progressive disease aim to slow down the disease progression and improve the quality of life [1]. Of note, effective treatment strategies to prevent PD are currently lacking. A cell therapy-based approach utilizing fetal tissue-derived neuronal cells with substantial dopamine production is being investigated for potential future technology for clinical translations [7]. The technical advancements, availability of fetal tissue, strict safety procedures and technical expertise are limiting factors for a cell therapy-based approach to deal with the increasing population with PD symptoms. Thus, the quest for a potentially feasible and widely applicable approach is gaining attention.

Among various strategies, plant-derived natural products have gained the considerable interest of scientists and pharmacologists for the treatment of several diseases, including neurodegenerative disorders [8]. Bioactive compounds from plant extracts have so far demonstrated great preventive and therapeutic potential. In particular, in the case of neurological disorders, phytoconstituents are known to generate various biological effects, including induction of growth factor, regulation of mitochondrial homeostasis, modulation of neurotransmitter metabolism and release, antioxidant and anti-inflammatory properties, etc. [8]. So far, various classes of phytochemicals, such as polyphenols, terpenoids, organosulfur compounds, nitrogen-based phytoconstituents, etc., have demonstrated decent therapeutic effects against various neurodegenerative diseases [9]. For example, polyphenols have exhibited significant antioxidant potential through free radical scavenging and chelating properties [10]. Various polyphenolic derivatives from plant extracts have demonstrated antiparkinsonian properties by inhibiting the production of inflammatory factors, including nitric oxide (NO), prostaglandin E2 (PGE2) and tumor necrosis factor-α (TNF-α), as well as reducing dopaminergic neuronal loss [11,12]. In this regard, Bu-7, a flavonoid derivative isolated from the leaf extracts of *Clausena lansium*, has shown a protective effect against the development of PD. The leaf extract increased the neural cell viability and reduced the apoptosis by suppressing the phosphorylation status and expression of mitogen-activated protein kinase (MAPK) protein families such as JNK and P38, which are crucial in the process of apoptosis in neural cells [13].

Pre-clinical models replicating disease features are widely used and are considered ideal tools for clinically relevant outcomes. In this regard, several studies have applied a variety of pesticides and routes of administration to replicate pathological and clinical findings of PD in animals [14]. These models demonstrate different amalgamations of the clinical and pathological characteristics of PD, including the selective loss of TH-positive neurons, the presence of LB in the SN, impaired striatal dopaminergic innervation or motor dysfunction, etc. [15]. Rotenone, a complex ketone pesticide naturally present in the roots of the Lonchocarpus plant, is used to induce motor dysfunctions in rodent models and replicates the PD features [16]. Rotenone, being lipophilic, can easily get through cell membranes and suppresses mitochondrial complex I, augmenting reactive oxygen species and, hence, PD etiology [17].

*Moringa oleifera* (MO) is a perennial tree with huge medicinal and nutritional benefits. It is found in northern India and Pakistan of the subcontinent South Asia. MO is cultivated in tropical and sub-tropical parts of the world as well. The seed extracts of MO show antioxidant properties in a cell-free medium [18]. Recent studies report the presence of bioactive peptides (such as –Napin-1A-P24565) from the crude extract of MO seeds [19]. A comparative study of MO seed extracts using water, ethanol and methanol as solvents revealed the presence of alkaloids, flavonoids, saponins, tannins and crude proteins in aqueous and ethanolic extracts [20]. Thus, the composition of plant extract largely depends on the solvent used and using extracts of the same plant with different solvents may lead to different therapeutic results. Similarly, a recent study reports over 250 compounds in two-dimension tandem mass spectrometry-based chemical analysis of MO seed extracts, pointing it to a potential source of bioactive compounds for therapeutic benefits [21]. The seeds of MO contain alkaloids, flavonoids, low-molecular-weight cationic proteins and methyl esters [22]. Previous research reports the free radical scavenging activities of aqueous, methanolic and acetone-derived MO seed extracts [21]. The year-round supply of MO seeds and the presence of bioactive compounds with antioxidant properties could provide protection to SN neurons of rotenone-induced PD mice and may reduce the extent of motor dysfunction. Therefore, the present study uses behavioral, biochemical and histological approaches to assess the beneficial effects of MO seed extracts on a rotenone-induced mice model of PD. This study reveals the protective potential of MO seed extracts on rotenone-mediated motor functional impairments in mice.

## 2. Materials and Methods

### 2.1. Plant Material

*M. oleifera* seeds were purchased from the local market of Lahore, Pakistan. The plant material was identified by the taxonomic experts (Dr. Zaheeruddin Khan) based on standard morphological characters, and a voucher specimen number (GC.Herb.Bot.1904) was submitted to Dr. Sultan Ahmad Herbarium (Government College University, Lahore Pakistan).

### 2.2. Chemicals

All the chemicals used were analytical grade: rotenone (Cat. # 241975, Oakwood Chemicals, Estill, SC, USA), ethanol (Cat. # 32221, RDH-Germany), DMSO (D1435, Merck, Lowe, NJ, USA), trichloroacetic acid (T6399, Merck), 2-thiobarbituric acid (T5500, Merck), nitrotetrazolium blue chloride (NDB0379, Bio Basic, Markham, ON, Canada), 1-chloro-2,4-dinitrobenzene (237329, Merck), Hematoxylin (H3136, Merck) and Eosin (Cat. # EE0190, Bio Basic).

### 2.3. Preparation of M. oleifera Seed Extracts

After proper identification of the plant materials, seed kernels of *M. oleifera* were ground to obtain seed powder without husk, and aqueous and ethanolic extracts of *M. oleifera* seeds were prepared according to the methodology described previously [18,23]. Briefly, 30.0 g of MO seed dry powder (obtained after removing the seed coat, and only the kernel part of the seed was ground and sieved to obtain it) was suspended in 300 mL of either autoclaved distilled water or absolute ethanol and adjusted in an orbital shaker at 200 rpm. Filtration was performed using Whatman No. 1 filter paper. The volume of the filtrate was reduced at 42 °C, and lyophilized extracts were stored at 4 °C until used. Final yields of 12.33% and 15.97% were obtained from aqueous and ethanolic extracts, respectively. The percentage yield of the extract was calculated using the following formula.

$$\mathrm{Percentage}\;\mathrm{Yield}=\frac{\mathrm{Final}\;\mathrm{extract}\;\mathrm{mass}}{\mathrm{Initial}\;\mathrm{mass}\;\mathrm{of}\;\mathrm{seed}\;\mathrm{powder}}\times 100$$

### 2.4. Experimental Design and Extract Administration

Adult male (8 weeks old) Swiss albino mice weighing 25–30 g were collected from the animal holding facility of Government College University, Lahore. Mice were housed in standard cages at 25 ± 2 °C, 12 h light and dark cycles, with unlimited supplies of rodent diet and water. All the procedures were performed following Institutional Bioethics Committee approval from Government College University Lahore, Pakistan (GCU-IIB-901). All procedures were performed according to the guidelines for the care and use of laboratory animals.

Mice were randomly assigned into 5 groups (*n* = 6 mice per group). The mice were subcutaneously injected with 2.5 mg/kg of rotenone to induce PD features [24]. The dosing schedule is given in Table 1. Before dosing schedule, the mice were acclimatized to laboratory conditions and trained for neurobehavioral assays, and baseline readings were taken. Behavioral experiments were performed before the start of dosing and on the 22nd day of trial (24 h after last dose).

### 2.5. Behavioral Tests for Motor Functions

The mice were subjected to six behavioral tests, namely open field test, stepping test, pole climb test, stride length test, beam walk test and tail suspension test, to examine PD motor symptoms. All the tests were videotaped and analyzed under controlled conditions.

Open field test investigates the locomotor activity of mice and is widely used to observe the motor dysfunction following onset of the PD symptoms [25]. Briefly, mouse was tagged with a black dot on neck scruff dorsally and videotaped in a 50 cm^2^ arena with a white paper sheet floor (marked in sixteen equal-sized squares). Mice were put in the center of the apparatus, and the mice were allowed to explore the apparatus for 5 min. After five minutes, the mouse was returned to its cage, and 70% ethyl alcohol was used to clean the open field apparatus. The total distance covered by mice in open field arena was estimated using ToxTrac software v. 2.96 with some modifications of the already reported article [26]. The number of entries in squares, marked on the white floor, was manually counted from video recordings of 5 min duration.

Stride length measurements reveal the extent of restricted locomotor function. Hence, the stride length determination evaluates the extent of locomotor dysfunction in PD mice [27]. A straight corridor (8.5 cm wide and 2.5 ft long with 8 cm high walls) was floored with a white sheet of paper. The footprints of inked paws of mice were taken on a white sheet of paper. The footprints were air-dried, and the distance between the inner toes of the two consecutive hind limbs after a stride was determined using a Vernier caliper. The three longest distances were measured, and the average value was analyzed.

In the current study, we used the tail suspension test to analyze the relative immobility of rotenone-treated mice. The apparatus consisted of a wooden base. It is 55 cm long, 60 cm tall and about 11.5 cm deep chambers. It consists of four chambers of the same size with a rod suspended 50 cm above the surface. To hold the mice on the rod, adhesive tape was used in such a way that the mice were in the middle of the apparatus and about 20–25 cm above the surface of the apparatus. The mice were suspended and filmed for 6 min with the aid of a video camera, as reported previously [28]. The severity of the disease was shown by the duration of immobility.

Beam walk test is widely used to assess motor function coordination [29]. Mouse was allowed to travel 1 m distance on an elevated beam (1 cm in diameter) held at 50 cm parallel to the ground. The mice were videotaped when crossing the beam. The maximum default value of 120 s was given to indicate the extent of the disease if the animal did not move or fell off the rod.

Pole test estimates the motor functional coordination and the extent of the slowness of the movement [30]. The apparatus consists of a vertical wooden rough-surfaced rod of 1 cm diameter and 50 cm tall. Mice were being carried at the top of the pole with their head facing upwards. The mouse turning over the top surface of the wooden rod and descending down the pole was videotaped. A default value of 120 s was applied if mouse failed to climb down or slipped.

Stepping test is used to assess the skill of walking and motor defects [31]. In this non-invasive test, the mouse is allowed to walk freely in a straight corridor of 1 m in length and videotaped for the time taken to traverse the distance.

### 2.6. Mice Brain Procurement

Mice were anesthetized with a cocktail of ketamine (100 mg/kg) and xylazine (10 mg/kg) intraperitoneally and were terminally euthanized by cervical dislocation. The brains were removed and dissected quickly and rinsed in ice-cold phosphate-buffered saline, and left hemisphere was separated and instantly frozen in liquid nitrogen and stored at −80 °C, while right hemisphere was fixed in 10% neutral buffered formalin (pH 7.0) for histopathology study.

### 2.7. Antioxidant Capacity Determination

The standard antioxidant markers were investigated from rotenone-inflicted mice PD brains vs. PD brains from MO seed extract-treated mice, as mentioned previously. Briefly, freshly procured mice brains were homogenized in a glass homogenizer. The homogenate was partially used for lipid peroxidation, and the remainder was suspended in phosphate buffer (0.1 M, pH = 7.4) and centrifuged (13,000× *g* at 4 °C for 30 min) to obtain post-mitochondrial supernatant (PMS).

Lipid peroxidation (LPO) measurement was performed by mixing 10% tissue homogenate with equal volumes of 0.67% trichloroacetic acid and 10% 2-thiobarbituric acid, boiled for 45 min in the water bath and centrifuged (6000× *g* for 10 min) as reported previously [32]. The absorbance at 532 nm was recorded from the separated supernatant. The LPO level was analyzed with the help of reactive compounds contained in the TBA. Using the extinction coefficient of 1.56 × 105/M/cm at 37 °C, it was demonstrated as μmol/g of tissue. The reduced glutathione (GSH) was quantified as described previously [33]. A total of 10% PMS (200 µL) was incubated with an equal volume of 4% sulfosalicylic acid at 4 °C for 1 h and centrifuged (1200 rpm for 15 min) to collect the supernatant. The supernatant, 10 mM DTNB (5,5′-dithiobis-2-nitrobenzoic acid) and sodium phosphate buffer (0.1 M, pH 7.4) were mixed, absorbance at 412 nm was recorded, and results are shown in nmol GSH/g of tissue. The activity of glutathione-S-transferase (GST) from PMS of mice brains was estimated using CDNB (1-chloro-2,4-dinitrobenzene) substrate as reported in a prior study [33]. The PMS (10% *w*/*v*), phosphate buffer (0.1 M, pH 7.4) and CDNB (1 mM) were mixed (2 mL final volume), and absorbance at 340 nm was noted. The GST activity is expressed as nmol/min/mg protein. The catalase (CAT) activity was determined from the reaction mixture containing phosphate buffer (0.1 M, pH 7.4), PMS (10%) and hydrogen peroxide (0.09 M). Change in color change was measured at 240 nm at an interval of 30 s for 120 s. The CAT activity was represented as nmol/min/mg of protein.

### 2.8. Histopathological Studies

The post-fixed right hemispheres of mice were processed under dissecting microscope to manually cut slices of −2 mm to −4 mm from Bregma to isolate the Substantia Nigra pars (SNc) compacta region, as described previously with some modifications [34,35]. The brain slices were embedded in paraffin, blocks were adjusted in microtome holder, and serial sectioning (5 µm thick) was performed. The brain sections were placed on glass slides and processed for Hematoxylin and Eosin staining, as reported previously [36]. The imaging was performed in camera-mounted light microscope, and photographs were taken.

### 2.9. Statistical Analysis

The data were analyzed, and result graphs were obtained using GraphPad Prism 5.0 (San Diego, CA, USA). The statistical differences (*p* < 0.05) were measured using one-way analysis of variance (ANOVA) followed by a Bonferroni post hoc test to compare all means pairwise. The data were represented as mean ± SEM.

### 2.10. Gas Chromatography−Mass Spectrometry (GC-MS) Analysis of Ethanolic Extracts of M. oleifera

GC–MS was performed on a Shimadzu GCMS-QP2010 single-quadrupole mass spectrometer equipped with a split–splitless injector, a model SPL-2010 Plus and a fused silica capillary column DB-5 (5% phenyl 95% dimethylpolysiloxane, 30 m × 0.25 mm i.d., film thickness 0.25 μm). The column was operated using an injector temperature of 200 °C and the following oven temperature profile: an isothermal hold at 50 °C for 3 min, followed by a ramp of 4 °C/min to 280 °C and an isothermal hold for 3 min.

Approximately 1.5 µL of ethanolic extracts of *M. oleifera* was injected using the split injection mode; the split flow ratio was 20:01. The helium carrier gas was flowed at 1.1 mL/min. The GC–TIC profiles and mass spectra were obtained using the Shimadzu LabSolution, All mass spectra were acquired in the EI mode (scan range of *m*/*z* 42–600 and ionization energy of 70 eV). The temperatures of the electronic-impact ion source and the MS quadrupole were 200 °C and 250 °C, respectively. The MSD transfer line was maintained at 280 °C for the analysis. The GC analysis was performed on a Shimadzu dual-channel gas chromatograph equipped with FTD detector using nonpolar columns under the same conditions as described above. The detector temperature was maintained at 300 °C for the analyses. Relative percentages of the compounds were calculated based on the GC–MS peak areas on DB-5 column without using correction factor and are reported in Table 2 according to their elution order on DB-5 column.

### 2.11. Identification of Bioactive Components from the Ethanolic Extract of M. oleifera

The identification of different components of ethanolic extracts constituents of *M. oleifera* was undertaken by matching their mass spectra with the library entries of mass spectra databases (NIST-library).

## 3. Results

### 3.1. MO Maintains Locomotor Activities

**Figure 1:**
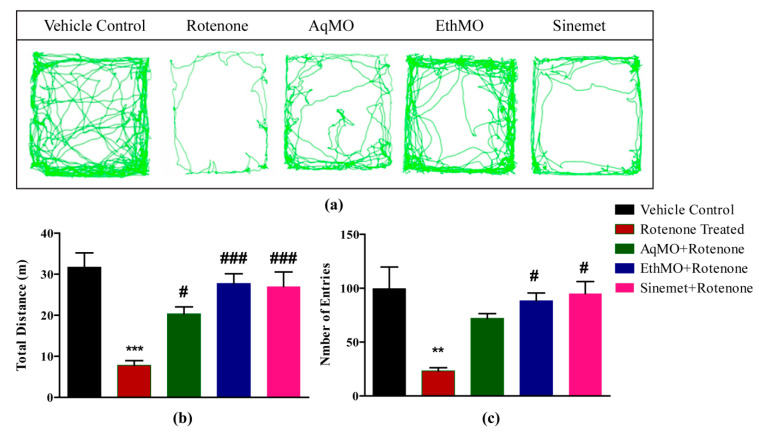
MO seed extracts maintain the exploratory activities of rotenone-inflicted mice. (**a**) Open-field trajectories of mice exploratory activity revealed a reduction in locomotor activity of mice in rotenone treatment group; however, a higher extent of locomotor activity was observed in MO extract-treated and Sinemet-treated groups. (**b**) A significant reduction in total distance (m) traveled by rotenone-treated mice compared to control was prevented significantly in extract-treated and Sinemet-treated groups. (**c**) The reduced number of entries in marked floor of open field was ameliorated in ethanolic MO seed extract and Sinemet groups. Data are represented as mean ± SEM; *n* = 6/group. ** *p* < 0.01, *** *p* < 0.001 vs. vehicle control; # *p* < 0.05, ### *p* < 0.001 vs. rotenone treated. One-way ANOVA followed by Bonferroni post hoc analysis.

Baseline behavioral assessment of mice after grouping without any treatment revealed the motor performance of all mice was parallel to their respective control group (Supplementary Figure S1). The ToxTrac trajectories revealed higher locomotor activity in control compared to rotenone-treated mice (Figure 1a). The mean distance covered in the open field arena was 31.7 ± 3.2 m in the vehicle control group, 7.82 ± 1.01 in the rotenone group, 27.76 ± 2.14 m in AqMO, 20.36 ± 3.2 m in EthMO and 27.02 ± 3.21 m in the Sinemet group. A significant (*p* < 0.05) longer distance was covered in both of the extract-treated groups compared to the rotenone-treated group (Figure 1b). Similarly, a higher number of entries in small squares of open field arena was observed in the extract-treated and Sinemet groups (Figure 1c).

**Figure 2:**
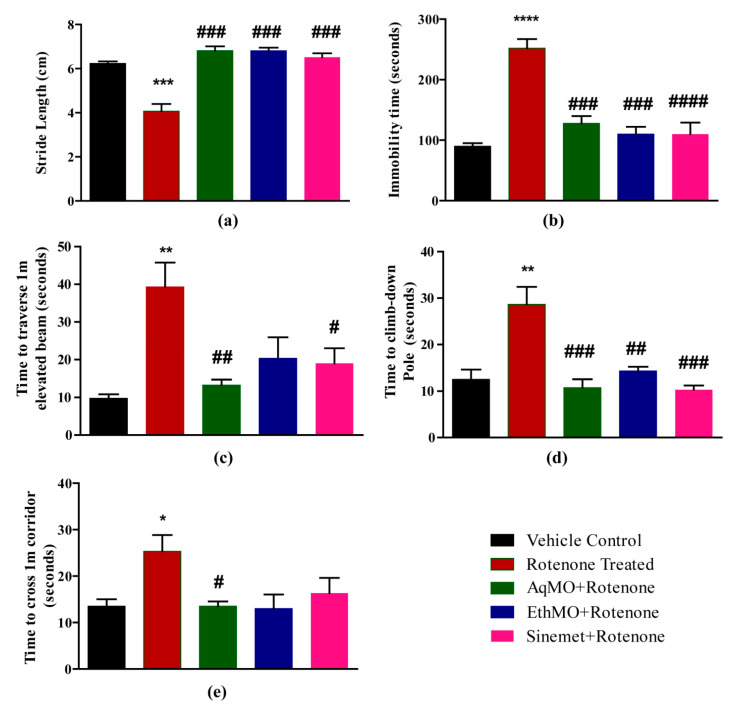
MO seed extracts protect against rotenone-mediated motor impairments. (**a**) Significant (*p* < 0.001) reduction in stride length of rotenone-treated mice compared to control mice was observed in MO extract-treated and Sinemet groups. (**b**) The increase in immobility in rotenone-treated mice was significantly prevented in extract-treated and Sinemet-administered groups. (**c**) The increased immobility time of rotenone-inflicted mice in tail suspension test was significantly prevented in extract-treated mice. (**d**) Extract-treated and Sinemet-administered mice climbed down the pole in significantly shorter times compared to rotenone-treated mice. (**e**) A significant reduction in delay to cross a 1 m straight corridor was observed in AqMO-treated mice compared to rotenone-administered mice, and a non-significant improvement was seen in EthMO- and Sinemet-treated mice. Data are represented as mean ± SEM; *n* = 6/group. * *p* < 0.05, ** *p* < 0.01, *** *p* < 0.001, **** *p* < 0.0001 vs. vehicle control; # *p* < 0.05, ## *p* < 0.01, ### *p* < 0.001, #### *p* < 0.0001 vs. rotenone-treated. One-way ANOVA followed by Bonferroni post hoc analysis.

Stride length measurements were 6.2 ± 0.09 cm in the vehicle control, 4.07 ± 0.32 cm in rotenone-treated, 6.8 ± 0.14 cm in AqMO, 6.8 ± 0.2 cm in EthMO and 6.51 ± 0.018 cm in Sinemet-treated mice. A significant (*p* < 0.001) reduction in stride length following rotenone treatment compared to the vehicle control was observed in the extract-treated and Sinemet-treated groups (Figure 2a). Similarly, the extent of immobility, while hung from the tail, was 90 ± 5.05 s in the vehicle, 252 ± 15.06 s in rotenone, 103.67 ± 5.93 s in AqMO, 115.25 ± 7.16 s in EthMO and 110 ± 19.1 s in Sinemet-treated mice. The extent of immobility in rotenone was augmented significantly (*p* < 0.001) compared to vehicle control, while the mice in treatment (AqMO, EthMO and Sinemet) groups had significantly reduced rotenone-mediated immobility (Figure 2b).

The mice traversed a 1 m distance on a beam held 50 cm above the ground in 9.75 ± 5.11 s in the vehicle, 36 ± 13.8 s in rotenone, 10.33 ± 1.20 s in AqMO, 9.5 ± 0.96 s in EthMO and 11.33 ± 1.20 s in Sinemet-treated mice. A significant (*p* < 0.01) increase in time in rotenone treatment reflects the delay in coordination functions compared to vehicle control. Of note, mice in the EthMO treatment and Sinemet treatment groups significantly retained motor coordination (Figure 2c). The mice demonstrated pole climb-down time of 14.33 ± 2.10 s in vehicle, 28.67 ± 3.76 s in rotenone, 14.5 ± 0.88 s in AqMO, 9 ± 1.8 s in EthMO and 10.33 ± 0.88 s in Sinemet treatment groups. Rotenone administration led to a significant (*p* < 0.01) increase in time taken to descent down; however, mice in treatment groups significantly (*p* < 0.01, at least) maintained agility compared to rotenone-inflicted mice (Figure 2d). A delayed completion of a 1 m straight corridor (25.33 ± 3.53 s) in rotenone-treated mice compared to vehicle control mice (13. ± 1.5 s) revealed compromised skill walking. However, mice in treatment (extracts and Sinemet) groups that covered the same distance in less time revealed a beneficial effect on skill walking (Figure 2e).

### 3.2. MO Effects on Oxidative Stress

**Figure 3:**
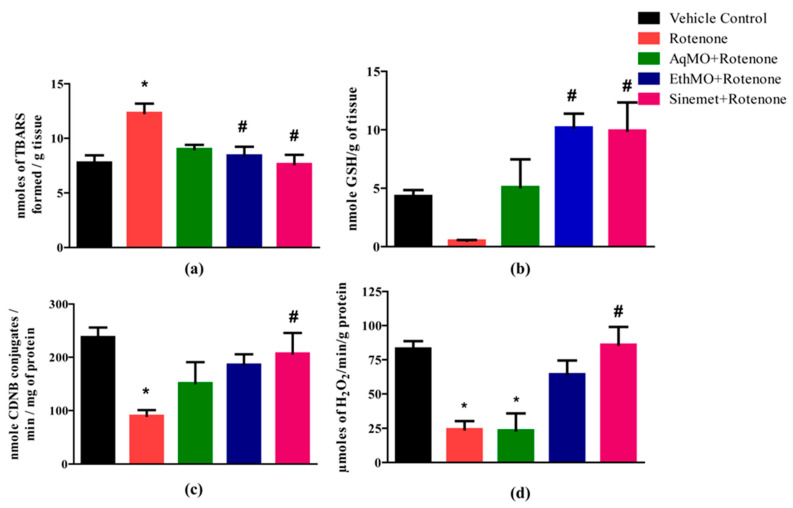
MO seed extracts partially restore the antioxidant capacities of brain of rotenone-treated mice. (**a**) A higher extent of lipid peroxidation in rotenone-treated mice was significantly prevented in EthMO- and Sinemet-administered mice groups. (**b**) The levels of reduced glutathione were considerably reduced in rotenone-treated mice brains, while a significant increase in reduced glutathione levels was observed in EthMO- and Sinemet-treated mice. (**c**) The activity of GST was significantly reduced in rotenone-treated mice, and a non-significant increase in GST activity was observed in MO extract-treated mice. (**d**) A sharp decline in CAT activity was observed in rotenone-treated and AqMO-treated mice; however, a sharp but non-significant increase in CAT activity was observed in EthMO-treated mice. Data are represented as mean ± SEM; *n* = 6/group. * *p* < 0.05 vs. vehicle control; # *p* < 0.0 vs. rotenone-treated. One-way ANOVA followed by Bonferroni post hoc analysis.

A significant (*p* < 0.05) increase in lipid peroxidation was observed in rotenone-treated mice compared to vehicle control; however, the extent of lipid peroxidation in extract-treated or Sinemet groups was not significant (Figure 3a). Rotenone treatment led to decreased levels of GSH, and the levels of GSH in EthMO and Sinemet groups were significant (*p* < 0.05) compared to rotenone control; however, a non-significant increase in GSH was observed in the AqMO-treated group (Figure 3b). GST activities were reduced in the rotenone (*p* < 0.05), AqMO (*p* > 0.05) and EthMO (*p* > 0.05) groups, while Sinemet treatment significantly reduced (*p* < 0.05) the GST activity compared to the rotenone group (Figure 3c). The CAT activity was significant (*p* < 0.05) in rotenone- and AqMO-treated groups; however, it was significantly (*p* < 0.05) maintained in the Sinemet group (Figure 3d).

### 3.3. Histological Findings

**Figure 4:**
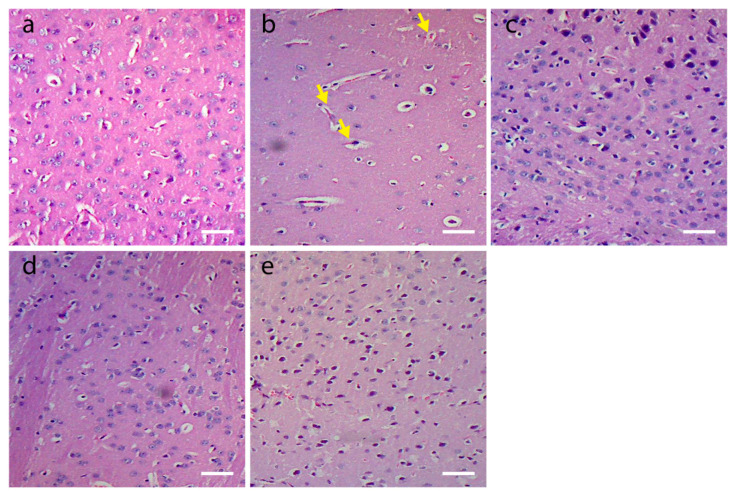
Histological investigation of Hematoxylin and Eosin-stained mice mid-brain sections (5 µm thick) viewed at 100× magnification under bright-field microscope. Scalebar: 100 µm. (**a**) Normal healthy neurons in control. (**b**) Reduced numbers of neurons in rotenone-treated mice with central chromatolysis (yellow arrows). (**c**) AqMO extract treatment prevents the loss of mid-brain neurons, as also seen in (**d**) EthMO extract-treated brain slices. (**e**) Sinemet treatment rescued the neuron loss in mid-brain of mice.

Brain sections from vehicle control mice demonstrated no histopathological changes (Figure 4a). A marked reduction in the number of neurons in the SN region and neurodegenerative signs, such as neuronophagia, compromised cell boundaries and central chromatolysis, were observed in rotenone-inflicted mice (Figure 4b). The background differences observed in H&E-stained tissue slices are evident owing to the technical challenges of processing the tissue at different times. The extent of neurodegeneration and neuron loss was less in AqMO and EthMO extract- and Sinemet-treated groups compared to the rotenone group (Figure 4c–e).

### 3.4. Phytochemical Characterization of EthMO Extract Using GC-MS Analysis

To determine the types of phytochemical constituents present in the seed extract of *M. oleifera* in ethanol (EthMO), the seed extract of *M. oleifera* (dried extract) was dissolved in ethanol and subjected to GC–MS analyses (Figure S1, Supplementary File). Notably, in this study, the biological potential of aqueous extract of MO was also evaluated against the rotenone-mediated motor function impairments in the PD mouse model. However, AqMO has demonstrated relatively low biological activity when compared to EthMO, and thus, it was not chosen for the detailed GC analysis. The spectroscopic analysis of the EthMO has led to the determination of a total of 10 phytochemical constituents, as shown in Table 2. The results have revealed that the EthMO was mainly dominated by 3-Ethoxycarbonyl-5-hydroxytetrahydropyran-2-one (entry 2 in Table 2), which was present in 15.96% of total phytochemical constituents. The other two compounds that are not far beyond the major constituent in terms of quantity are Di-*n*-octyl phthalate and 3-Pyrrolidinol, which are present in 14.9 and 14.5%, respectively. In addition to these compounds, hexadecanoic acid, 2,3-bis[(trimethylsilyl)oxy]propyl ester, is also present in significant amounts (11.4%), while the rest of the compounds were merely present in between 2 and 9% of the total amount of substances.

## 4. Discussion

**Scheme 1:**
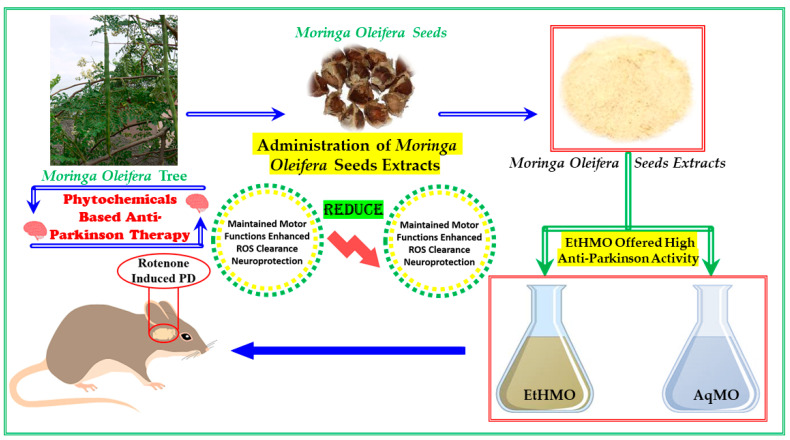
Schematic representation of *M. oleifera* seed extracts and their anti-Parkinson potential.

In the current study, we aimed to explore the effects of aqueous and ethanolic extracts from *M. oleifera* seeds on a rotenone-induced PD mouse model (Scheme 1).

Lack of muscle coordination, reduced exploratory behavior and compromised agility and balance are the rotenone-mediated motor dysfunctions that were reported earlier [1]. The open field, stride length measurements, tail suspension, beam walk, pole climb-down and stepping tests are widely used to assess motor dysfunctions in PD pre-clinical models [24,25,37]. Collectively, these tests assess exploratory activities, walking, motor coordination, balance, agility, restricted locomotion and immobility. In the current study, rotenone-induced PD mice following MO extract treatments or Sinemet administration demonstrated better exploratory activities compared to only rotenone-induced PD mice in open field tests. This finding suggests that the beneficial role of extracts in maintaining normal locomotor functions and performance of vehicle control mice is comparable to that of the previous report [38]. 

The stride length measurements unveil the walking alterations characteristics and reflect the normal vs. impaired gait functions of the mice. In the current study, the reduction in stride length of rotenone-induced PD mice was in line with a previous study [39]. The failure to shorten the stride length in MO seed extract-treated groups was comparable with the Sinemet group, suggesting a comparable efficiency of extracts with the standard drug applied for initial PD treatment. Tail suspension test unveiled the delay in motor functional impairments in MO extract-treated groups compared to rotenone treatment, as rotenone-mediated immobility and anxiety-like behavior were prevented in treatment groups, as reported in PD models previously [40]. The beam-walk test enabled us to evaluate the motor coordination and balance in rotenone-exposed PD mice. The ethanolic MO extract-treated mice demonstrated significantly better coordination compared to rotenone-treated mice, suggesting a maintained motor coordination and limited PD progression. The prevention of motor coordination dysfunction in MO extract treatment was further investigated using the pole climb-down test. Mice demonstrated a significant difference compared to the rotenone-treated group in terms of time taken to descend down from the vertical pole. Thus, in line with previous studies, pole climb-down results reflect the normal functioning of basal ganglia in MO extract-treated mice and in the Sinemet-treated group [41].

Oxidative stress is known to cause alterations in iron, ferritin and metallic ion concentrations in dopaminergic neurons of SN [42]. Of note, oxidative stress-induced nuclear translocation of nuclear factor-KB increases the risk (up to 70-fold) of dopaminergic cell death in PD [43]. Rotenone-induced mitochondrial complex I dysfunction is the primary means of oxidative stress reported to trigger toxicity of intact dopaminergic neurons [44]. Thus, the investigation of the antioxidant potential of mice brains exposed to rotenone and extract treatments is perhaps the most relevant method to assess the protective potential of tested drugs modulating the oxidative status of cells. We explored the well-known antioxidant markers, reduced glutathione, glutathione-S-transferase and catalase from mouse brain homogenates. In the current study, the gross lipid peroxidation level of rotenone-induced mice brains was elevated, showing decreased antioxidant potential; however, a significant reduction of LPO in ethanolic MO seed extract-treated and Sinemet-treated mice depicted the augmented antioxidant capacities to clear free radicals. The depleted levels of reduced glutathione (GSH) are reported from SN of PD post-mortem brains [45]. A recent study reported that GSH administration improves the motor functions of PD rats [46], suggesting a potential role as a scavenging agent to neutralize oxidative stress. We observed a huge increase in GSH levels in ethanolic MO- and Sinemet-treated mice. It has been reported that suboptimal (30–50%) levels of GSH in PD are due to suboptimal production [47]. Of note, an increased level of GSH is observed in the current study, suggesting that MO ethanolic extract seems to protect GSH production in rotenone-induced PD mice. The antioxidant enzyme glutathione-S-transferase (GST) clears the byproducts of lipid peroxidation and protects the dopaminergic neurons in PD models [48]. Environmental toxin rotenone exposure led to decreased activities of GST; however, non-significant increases in GST activities were observed in MO seed extract-treated mice, suggesting a moderate influence of extracts on PD mice. A previous study reports the neuroprotection of dopaminergic neurons through increased GST activity [49]. The augmented GST activity in MO extract-treated mice suggested a possible involvement in neuroprotection of rotenone-induced degeneration of the mid-brain. The catalase (CAT) activity involves the elimination of oxygen free radicals, and a significantly decreased CAT activity in rotenone-treated mice suggested a perturbed homeostasis of free radicals. A non-significant increase in ethanolic MO extract was noticed; however, no change in the CAT activity of aqueous MO extract was observed. It suggests the non-involvement of extract treatment in the mediation of oxidative stress through CAT means.

The improved motor functions and higher antioxidant capacities in extract-treated groups suggested the possible neuroprotection of the mid-brain neurons. Rotenone treatments led to the degeneration of SN neurons in PD animal models [50]. In our study, the extent of neurodegeneration in rotenone-inflicted mice treated with/without MO extracts was investigated. Consistent with prior studies [51,52], mid-brain sections of rotenone-treated mice demonstrated eosinophilic lesions (yellow arrows), necrotic changes and shrunken cytoplasm. Although brain sections show some background differences, the difference in cell density in rotenone-treated mice and MO treatment groups is quite obvious. Consecutive administration of MO extracts attenuated the neurodegenerative changes and hence maintained the characteristic shape of the neurons.

## 5. Conclusions

The MO seed extract administration to rotenone-challenged adult mice demonstrated maintained motor functions as assessed through a battery of neurobehavioral tests. The mice brain homogenates following in vivo MO seed extract treatment completion depicted significant antioxidant potential through augmented levels of reduced glutathione, glutathione S transferase and catalase activities compared to PD mice. Histological investigations unveil that MO seed extract treatment imparts strong neuroprotection in the mid-brain of mice. GC-MS profiling of MO seed extract (EthMO) reveals a bouquet of biologically active ingredients. In the current study, the prevention of motor dysfunctions in MO seed extract-treated mice under rotenone exposure seems to be possibly through the antioxidant potential of the extract ingredients. The findings of the current study suggest that future studies should be conducted to explore the single active ingredient from the extract with possible neuroprotective capacities. Collectively, the current data point to the therapeutic potential of MO seed extracts with possible clinical applications.

## Figures and Tables

**Table 1:** Animal grouping and treatment schedule.

Animal Group Title	Treatment	Duration
1. Vehicle Control	DMSO containing sunflower oil treated (1 mL/kg) + distilled water, oral administration (10 mL/kg)	21 days (once a day)
2. Rotenone Control	Rotenone (2.5 mg/kg) in 2.5% DMSO, subcutaneous administration (1 mL/kg) + distilled water, oral administration (10 mL/kg)	21 days (once a day)
3. EthMO Seed Extract Treated	Rotenone (2.5 mg/kg) in 2.5% DMSO containing sunflower oil, subcutaneous administration (1 mL/kg) + 200 mg/kg EthMO extract, oral administration (10 mL/kg)	21 days (once a day)
4. AqMO Seed Extract Treated	Rotenone (2.5 mg/kg) in 2.5% DMSO, subcutaneous administration (1 mL/kg) + 200 mg/kg AqMO extract, oral administration (10 mL/kg)	21 days (once a day)
5. Sinemet Control	Rotenone (2.5 mg/kg) in 2.5% DMSO, subcutaneous administration (1 mL/kg) + Sinemet (20 mg/kg) in distilled water, oral administration (10 mL/kg)	21 days (once a day)

**Table 2:** Chemical compounds identified from the ethanolic seed extract of *M. oleifera*.

Peak#	R. Time	Compounds	M.F.	M.W.	CAS No.	EthMO (%)
1	6.582	3-Pyrrolidinol	C_4_H_9_NO	87	2799-21-5	14.5
2	8.301	3-Ethoxycarbonyl-5-hydroxytetrahydropyran-2-one	C_8_H_12_O_5_	188	-	15.9
3	11.794	Diethyl Phthalate	C_12_H_14_O_4_	222	84-66-2	8.8
4	14.190	Hexadecanoic acid, methyl ester	C_17_H_34_O_2_	270	112-39-0	2.5
5	14.268	(9Z)-8-Methyl-9-tetradecenoic acid	C_15_H_28_O_2_	240	457653-87-1	3.8
6	16.465	Hexadecyl 2,2,3,3,3-pentafluoropropanoate	C_19_H_33_F_5_O_2_	388	6222-7-7	8.3
7	17.437	Hexadecanoic acid, 2,3-bis[(trimethylsilyl)oxy]propyl ester	C_25_H_54_O_4_Si_2_	474	1188-74-5	11.4
8	17.823	Hexadecanoic acid	C_16_H_32_O_2_	256	57-10-3	7.0
9	18.203	Octadecanoic acid	C_18_H_36_O_2_	284	57-11-4	3.0
10	18.771	Di-*n*-octyl phthalate	C_24_H_38_O_4_	390	117-84-0	14.9
Total Identified						90.1

R. Time = retention time; M.F. = molecular formula; M.W. = molecular weight.

## Data Availability

Data are contained within the article.

## Supplementary Materials

The following supporting information can be downloaded at https://www.mdpi.com/article/10.3390/plants13111479/s1. Figure S1: GC-MS analysis of ethanolic extract of *M. oleifera* seeds. Identified peaks are numbered according to the Table 2.
