# Supplementary material for: In Vivo Study of Moringa oleifera Seed Extracts as Potential Sources of Neuroprotection against Rotenone-Induced Neurotoxicity
Source: Plants (Basel). 2024 May 27;13(11):1479. doi: 10.3390/plants13111479 (PMC11175126; doi:10.3390/plants13111479)
Supplement: Supplementary file 1 [file plants-13-01479-s001.zip › plants-3024263-supplementary.pdf]

plants

Supplementary File

In Vivo Study of *Moringa oleifera* Seed Extracts as Potential Sources of Neuroprotection against Rotenone-Induced Neurotoxicity

Chand Raza [1,*], Sehrish Mohsin [1], Mehwish Faheem [1], Uzma Hanif [2,*], Hamad Z. Alkhathlan [3], Mohammed Rafi Shaik [3], Hasib Aamir Riaz [4], Rabia Anjum [1], Husna Jurrat [1] and Merajuddin Khan [3,*]

[1] Department of Zoology, Government College University, Lahore 54000, Pakistan
[2] Department of Botany, Government College University, Lahore 54000, Pakistan
[3] Department of Chemistry, College of Science, King Saud University, P.O. Box 2455, Riyadh 11451, Saudi Arabia
[4] Department of Molecular Biology, Cell Biology and Biochemistry, Brown University, Providence, RI 02912, USA
* Correspondence: chandraza@gcu.edu.pk (C.R.); uzmahanif@gcu.edu.pk (U.H.); mkhan3@ksu.edu.sa (M.K.); Tel.: +966-11-4675910 (M.K.)

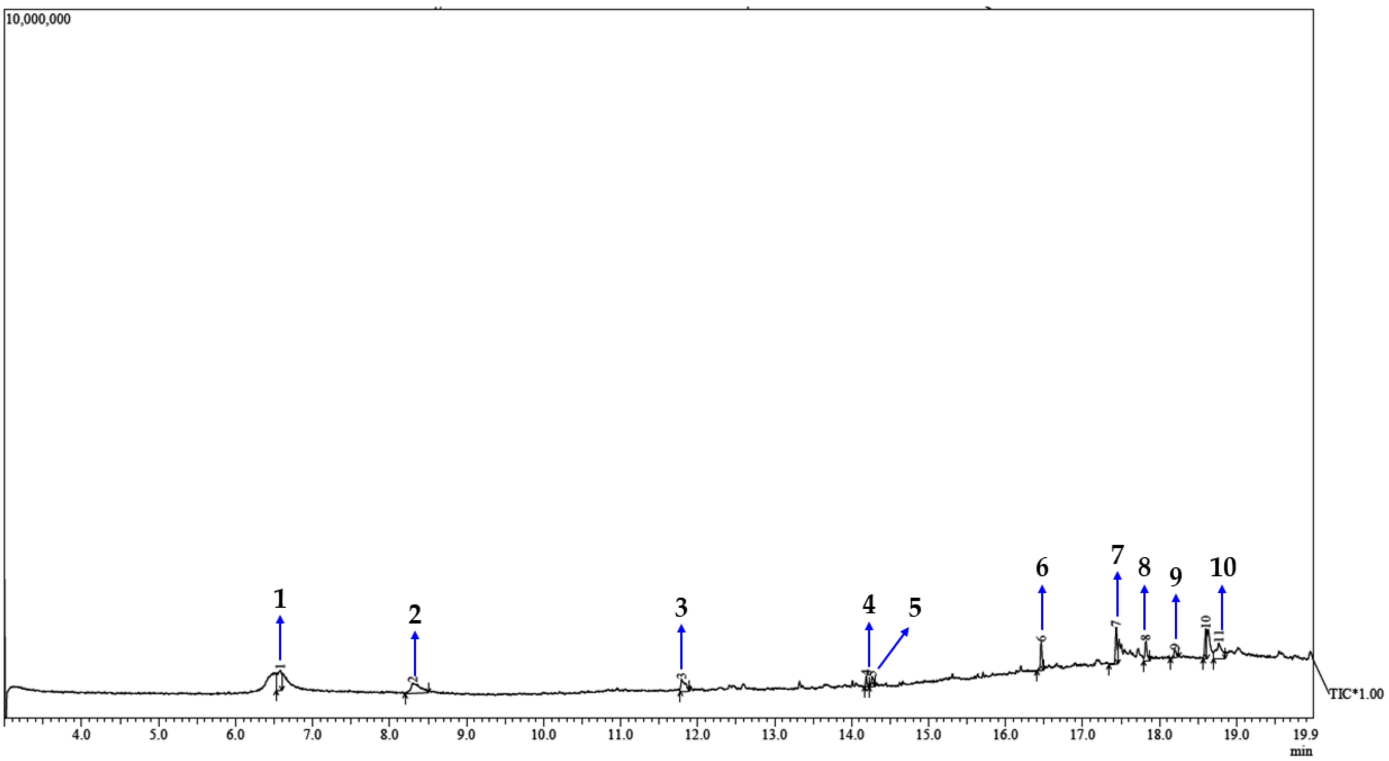

Figure S1. GC-MS analysis of ethanolic extract of *M. oleifera* seeds. Identified peaks are numbered according to the Table 2.
